# Related Factors and Treatment of Postoperative Delirium in Old Adult Patients: An Integrative Review

**DOI:** 10.3390/healthcare9091103

**Published:** 2021-08-26

**Authors:** Carlos Méndez-Martínez, María Nélida Fernández-Martínez, Mario García-Suárez, Santiago Martínez-Isasi, Jesús Antonio Fernández-Fernández, Daniel Fernández-García

**Affiliations:** 1 Department of Nursing and Physiotherapy, University of León, 24071 León, Spain; mgars@unileon.es (M.G.-S.); jfernf@unileon.es (J.A.F.-F.); dferg@unileon.es (D.F.-G.); 2 University Hospital of León, 24071 León, Spain; 3 Department of Biomedical Sciences, Institute of Biomedicine (IBIOMED), Veterinary Faculty, University of Leon, 24071 Leon, Spain; mnferm@unileon.es; 4 CLINURSID Research Group, Psychiatry, Radiology, Public Health, Nursing and Medicine Department, Universidade de Santiago de Compostela, 15705 Santiago de Compostela, Spain; santiago.martinez.isasi@udc.es; 5 Simulation and Intensive Care Unit of Santiago (SICRUS) Research Group, Health Research Institute of Santiago, University Hospital of Santiago de Compostela CHUS, 15706 Santiago de Compostela, Spain

**Keywords:** integrative review, critical care, delirium, postoperative delirium

## Abstract

“Postoperative delirium” is defined as delirium occurring in the hospital up to one week after a procedure or before discharge (whichever occurs first) that meets the DSM-5 diagnostic criteria. Objectives: To describe the risk factors related to this pathology and identify effective non-pharmacological forms of treatment. An integrative review of the available literature was performed. The search results considered included all quantitative studies published between 2011 and 2019 in both English and Spanish. A total of 117 studies were selected. Advanced age was identified as the principal risk factor for postoperative delirium. Nursing interventions appear to be the key to preventing or reducing the seriousness of delirium after an anaesthetic episode. The aetiology of postoperative delirium remains unknown, and no treatment exists to eliminate this pathology. The role of nursing staff is fundamental in the prevention, diagnosis, and management of the pathology.

## 1. Introduction

“Postoperative delirium” is defined as delirium that occurs in the hospital up to one week after the procedure or before discharge (whichever occurs first) that meets the DSM-5 diagnostic criteria. Factors to consider in postoperative delirium include previous drug use, alcohol use, dementia or previous delirium, persistent effects of drugs, metabolic abnormalities, hypoxia, electrolyte imbalance, and infection [1]. The condition is characterized by changes in cognitive function (memory impairment, disorientation, agitation, and/or confused speech) and in the level of consciousness (alternating periods of alert and periods of delirium) [2]. Onset is sudden, fluctuating, and reversible; it cannot be explained by pre-existing neurocognitive disorders or as a sign that the patient is developing such a disorder, and it manifests shortly after surgery (within days or hours) [2,3,4].

Emergence delirium shares certain similarities to postoperative delirium, and although it does not have a clear definition, it shares some risk factors with postoperative delirium: both can be triggered by noxious stimuli during the perioperative and postoperative periods, but they are different phenomena [5]. Surgical patients may experience emergence delirium initially, which later progresses to postoperative delirium. While emergence delirium is more prevalent in healthy pediatric patients and young adults, postoperative delirium usually affects older patients with multiple comorbidities. The two phenotypes are often referred to as synonymous in the literature, but it is important to differentiate them, as their prevention and treatment strategies differ. While emergency delirium requires pharmacological treatment, postoperative delirium can benefit from both pharmacological and non-pharmacological treatment, such as time–space orientation [5].

While delirium has other causes, treatments, and clinical significance, postoperative delirium is associated with a delay in postoperative recovery, increased costs, and increased morbidity and mortality. Delirium “in general” and “emergency delirium” have been widely studied, while despite a large number of articles published on postoperative delirium, there is inadequate evidence regarding its prevention and management, in terms of both pharmacological and non-pharmacological treatment [5,6,7].

Patients with postoperative delirium demonstrate changes in attention levels, showing difficulty focusing, maintaining, or directing attention. In addition, perception is altered such that the patient may misinterpret reality and have delusions or hallucinations which, in turn, affect their behaviour. This may be expressed as fear or aggression towards external stimuli [2,6,8,9]. The patient usually begins with spatio–temporal disorientation, an increase or decrease in psychomotor activity, and disturbances in the sleep–wake cycle [10]. The patient will also present with psychomotor agitation and disorientation which, in general, alternates with episodes of hypersomnia [10]. Patients have periods of lucidity, generally in the morning, with the maximum level of disturbance occurring at night. Unknown environments or those with few external stimuli aggravate the patient’s situation [10,11].

The physiopathology of postoperative delirium is currently unclear; its onset has been correlated with several factors, but it is by no means fully defined. Some of these factors include age, sex, preoperative state of health, type and duration of surgical intervention, previous drug consumption, hypotension, time spent in cardiac bypass during heart surgery, presence of inflammation, presence of stress hormones, ischemia, hypoxemia, certain neurotransmitters, water–electrolyte balance, pain, and nutritional status, among others [12,13,14,15,16,17,18,19]. Because the pathophysiology of postoperative delirium is not exactly known, it is important to know its predisposing factors (vulnerability of the patient; that is, factors that are present in many of the patients who develop it) as well as the precipitating factors (factors, generally external, that facilitate the occurrence of postoperative delirium). Having knowledge of both types of factors could help in the prevention and management of postoperative delirium [10,14,15].

The current reported incidence of postoperative delirium in reference to old adults is underestimated [20]. It varies significantly as a function of the age of the patient, their preoperative status (according to the ASA classification system), whether surgery is planned or emergency, the type and duration of surgery, the diagnostic methods used, and the criteria used [1,21,22,23,24,25,26,27,28]. At least two of every three cases of delirium occur within the first postoperative day, with peak onset occurring on the second postoperative day. A delayed onset of postoperative delirium is associated with significant postoperative complications or withdrawal from alcohol or drugs, according to Espinosa Calderón et al. [29].

Postoperative delirium can be classified clinically as a hyperactive, hypoactive, or mixed presentation. The hyperactive form is a subtype of delirium in which there is increased sympathetic nervous system activity, causing symptoms such as oversensitivity to stimuli, perceptual disturbances, psychomotor overactivity, auto-aggression, or physical and verbal aggression towards health-care staff [30]. Hypoactive delirium is characterized by a diminished response to stimuli, hypersomnia, bradypsychia, and lethargy. There is a high probability of this condition remaining undiagnosed unless cognitive changes are specifically tested for. Lastly, mixed delirium combines the characteristics of the hyperactive and hypoactive types [30,31,32,33].

The most common form of delirium is the hypoactive type. McDaniel et al. [34] established its incidence as 50%, with that of the hyperactive and mixed forms given as 25% in both cases. Rodríguez [11] stated that hyperactive delirium has an incidence of 5%, whereas the mixed and hypoactive types both have incidence rates of 45%. Guenther et al. [35] stated that the hyperactive form has an incidence of 10%, and the hypoactive type has an incidence of more than 50%. Lastly, Bettelli and Neuner [35] stated that mixed delirium has the highest incidence, 50%, with the hyperactive and hypoactive forms having incidences of 10–30% and 20–40%, respectively.

Postoperative delirium is associated with a multitude of complications, such as a longer length of stay in intensive care units [19,36,37] and longer hospital stays [6,38,39,40,41], as well as a higher cost per patient [7,41,42,43,44,45], a high incidence of subsequent discharge to nursing homes [23,46,47], and a greater likelihood of rehospitalization [6,14]. The pathology contributes to the appearance of cognitive changes [45,48] and permanent deterioration in function [37,49,50,51] and has been shown to be a precursor to dementia [11,33,40,42,52]. In the immediate postoperative period, the condition can also give rise to complications such as accidental removal of intravenous lines, haemorrhage at the site of surgery, and even aggression towards nursing personnel [21]. It is also associated with a potential risk of sepsis [22,53] and an elevated rate of morbidity [46,48,49,54,55,56,57,58,59].

Over the years, a series of postoperative delirium assessment scales have been developed for use by nursing staff in anaesthesia recovery rooms and intensive care units in the immediate postoperative period. Among these, several stand out, including the Confusion Assessment Method (CAM) [16,19,42]; the Confusion Assessment Method—Intensive Care Unit (CAM-ICU) [50,60,61,62]; the Nursing Delirium Screening Scale (NuDESC) [24,60,61], and the Intensive Care Delirium Screening Checklist (ICDSC) [27,63]. This list is not exhaustive and other scales do exist, such as the Delirium Rating Scale Revised-98 [27].

Since nursing staff are usually the first to observe changes in the behaviour of patients, it is very important that these professionals are able to identify postoperative delirium early so that the pathology can be treated and complications avoided in the short- and long-term [64].

It must be emphasized that there are no existing treatments, either pharmacological or otherwise, that can completely eliminate the risk of postoperative delirium. Nevertheless, the literature does identify certain pharmaceuticals and interventions that alleviate it. Interventions most often used by nursing professionals include placing clocks and calendars in highly visible locations to help the patient regain spatio–temporal orientation, encouraging family members to visit, designing non-drug-based schedules to promote good-quality sleep, clear differentiation between night and day, elimination of nuisance noise at night, allowing the use of hearing aids and glasses to avoid audio–visual deficit, monitoring liquid intake to ensure an optimal water–electrolyte balance, avoiding malnutrition, and the evaluation and active treatment of pain [17,65].

In terms of research questions and objectives, this review integrates theory and empirical evidence concerning postoperative delirium in the context of postoperative recovery (Post Anaesthesia Care Unit) and intensive care units in order to establish a framework to support nursing interventions aimed at reducing the incidence of this condition. Because postoperative delirium has a higher incidence in the old adult population, this study focuses on this age group.

To this end, we propose the following objectives related to postoperative delirium: in the first instance, we aim to determine the major factors associated with its onset and development and, in the second instance, we aim to outline the principal treatment methods, distinguishing nursing interventions from pharmacological interventions while also determining which drugs have been proven to be useful in the treatment of postoperative delirium and which have not, be studied in the context of post-anaesthesia care units and intensive care units.

## 2. Materials and Methods

An integrative review was completed, as it was felt that this was the most appropriate method for summarizing and analysing both empirical and theoretical literature in a systematic fashion such that the results would have direct applicability to clinical practice. 

The bibliographic search and selection of articles were completed in accordance with PRISMA (Preferred Reporting Items for Systematic Reviews and Meta-Analyses). To assess the methodological quality of the included articles, the assessment guidelines established in the John Hopkins Nursing Evidence-Based Practice Model were used [66].

### 2.1. Search Strategy

The literature search was completed between the months of May and June 2021. The scientific databases used for this search were PubMed, Cochrane, Google Scholar, SciELO, CUIDEN, and CINAHL.

We used search terms from the Medical Subject Headings (MeSH) and the Descriptores de Ciencias de la Salud (DeCS), in both English and Spanish. These included “delirium” (D003693), and “Emergence delirium” (D000071257); the related search terms “Postoperative Delirium”, “Anaesthesia delirium”, and “surgery delirium”; additionally, their corresponding Spanish translations. 

For the purposes of the search, related terms were linked using the Boolean operator “OR”, while in order to link different concepts the Boolean operator “AND” was used. 

As an example of a search strategy, an example of a PubMed search is shown. The filters used were “All types of articles”, “Full text”, “Since 2011”, “Human”, “Not pediatric”, and “Not children”, and the search term was “postoperative delirium”.

Another example is a Cochrane search that was performed with the limits “all content types”, “Cochrane Library Publication date between 2011 and 2019”, “CENTRAL Trials only Original publication year between 2011 and 2019”, and “Variations of search words”.

A search in Google Scholar was performed with the filters “Articles”, Specific Interval “2011–2019”, “Include patents”, and “Include citations”

A search in SciELO was performed with the filters “Language: Spanish”, “Language: English”, and “Year of publication: 2011–2018”.

A search in CUIDEN was performed with the filter “years between 2011 and 2019”.

The last example was a search performed in CINAHL with the filters “Search mode: Boolean/phrase, apply related words”, “Full text”, “Abstract available”, “English language”, “Human”, “Publication subset: all”, “Publication type: all”, “Topics of interest: all”, “Language: all”, “Exclude medline records”, “ Randomized controlled trials”, “Sex: all”, “Age groups: all adult”, and “Full text in PDF”.

### 2.2. Selection Criteria

Articles selected for this study complied with the following criteria: all articles were published between 2011 and 2021 with the aim of selecting only the most recent articles on postoperative delirium. The reason for starting the search in 2011 was because there were two high-quality reviews on the subject covering articles published up until 2010 [65,66]. Articles published in English and Spanish were included. All quantitative studies were included, and full-text versions were obtained; randomized clinical studies, systematic reviews, integrative studies, and bibliographic reviews were included. Descriptive studies with the goal of synthesizing and giving an improved picture of the literature published to date were also included. The population included in our study comprised old adult patients (aged over 65 years old, because this is the age group associated with a higher incidence of delirium) of both sexes requiring admittance to a recovery or intensive care unit immediately after surgery. Regarding the results, the articles selected combined information about associated factors, treatment (pharmacological as well as non-pharmacological), nursing interventions to reduce the incidence of postoperative delirium, and methods used for diagnosis. 

All articles that did not meet these criteria were excluded.

### 2.3. Article Selection and Data Abstraction

Initially, in order to minimize the risk of bias, the literature search was completed by two independent reviewers using the aforementioned databases, and the search criteria were outlined. Once the duplicates had been eliminated, the first selection of articles was completed through an independent analysis of article titles.

Following on from this, a second review was completed. This involved reading the abstracts and keywords of the articles selected by the initial two reviewers. In this way, the final selection of articles was composed. Any discrepancies were discussed and a solution found by consensus between reviewers.

Once the full texts of the articles had been obtained, articles were put through a third and final selection process in which articles to be included in this review were chosen.

Finally, once the final selection of articles for inclusion in this review was determined, the following data were extracted from each article: date of publication, title of article, type of study, population, type of intervention, intervention/variables of results, results, influencing factors, influencing pharmaceuticals, pharmaceuticals that reduced the incidence of the condition, non-pharmacological interventions, and diagnostic tests/scales used. This was conducted with the aim of synthesizing all of the information gathered and facilitating its management.

Given that the studies reviewed were very heterogeneous and used many different interventions and forms of evaluation, it was not possible to perform a meta-analysis.

### 2.4. Characterisation of Articles

**Figure 1:**
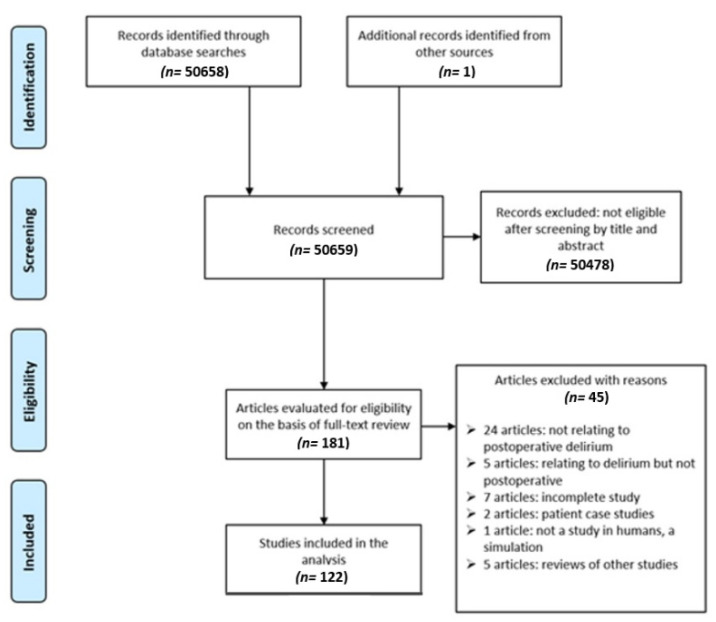
PRISMA flow chart showing the results of the search completed.

Figure 1 shows a PRISMA flow chart of the results obtained from the search process. In the initial search, a total of 50,658 results were obtained. In total, 181 articles fulfilled the selection criteria, and full-text versions of these were obtained. After a full analysis, 45 articles were excluded for the following reasons: 24 were not related to postoperative delirium, 5 were related to delirium types other than postoperative delirium, 7 studies were not complete at the time of access, 1 was a qualitative study, 2 articles concerned case studies of particular patients, 1 article involved a simulation rather than a study on humans, and 5 articles were reviews of other articles.

The final selection included 122 articles with the following distributions: 4 case–control studies, 22 randomized clinical trials, 2 analytic studies, 1 quasi-experimental study, 12 cohort studies, 7 descriptive studies, 37 observational studies, 1 case series, 2 meta-analyses, 31 bibliographic reviews, 1 integrative review, 10 systematic reviews, and 6 systematic reviews with a meta-analysis included.

### 2.5. Evaluation of Methodological Quality

In order to evaluate the methodological quality of the articles included in our study, we used the evaluation guidelines set out in the John Hopkins Nursing Evidence-Based Practice Model: a level was assigned to the evidence (I, II, III, IV, or V) and a quality rating of either A (high quality), B (good quality), or C (low quality, or having significant defects) was determined based on the study design and quality of the evidence contained in each article [66].

**Figure 2:**
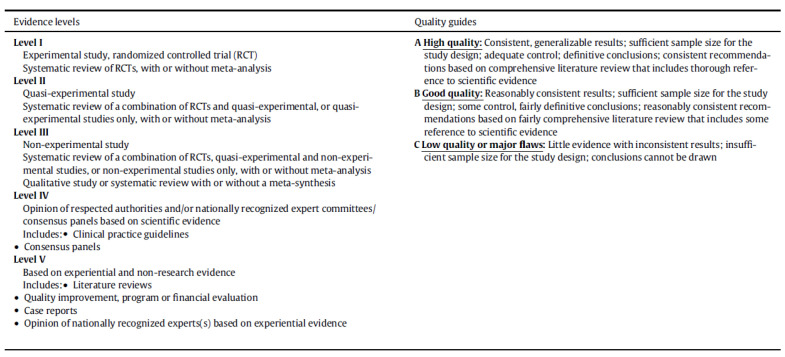
Evaluation of studies according to the John Hopkins Nursing Evidence-Based Practice Model. Levels of evidence and quality.

Figure 2 shows a table explaining the levels of evidence and the quality ratings proposed in the John Hopkins Nursing Evidence-Based Practice Model [66].

## 3. Results

### 3.1. Factors Associated with the Onset and Development of Postoperative Delirium

Table 1 presents the predisposing factors associated with the risk of a patient developing postoperative delirium. Table 2, on the other hand, lists the triggers that affect the patient. Although the pathophysiology of postoperative delirium occurring after an anaesthetic episode is still unknown, many factors were associated with its appearance and development. We divided these factors into two groups, which are shown in the following tables with the items ordered according to their level of evidence and quality (John Hopkins Nursing Evidence-Based Practice [66]) according to the studies from which they were extracted.

Studies suggest that the most important predisposing factors to consider in the development of postoperative delirium are advanced age, pre-existing cognitive deterioration, pain, elevated ASA score, alcohol abuse, hypoxemia, metabolic disturbances/water–electrolyte imbalances, vision impairment, and auditory impairment. On the other hand, the use of benzodiazepines, cardiac surgery, heart-lung bypass, the duration of surgery, emergency surgery, orthopaedic surgery, the type of surgery, receiving a major transfusion, and being admitted to ICU were described as factors predisposing an individual to its development.

### 3.2. Principal Methods of Treatment

Currently, prevention is perhaps the most efficient and cost-effective way to treat delirium at the time of initial presentation. The role of nursing personnel is key to the prevention and identification of delirium and in terms of implementing non-pharmacological interventions to treat it. 

Although there is still no established “gold standard” for the treatment of postoperative delirium, studies have suggested numerous non-pharmacological and effective pharmacological interventions that can reduce the symptoms of this pathology. We consider that for an intervention to be effective, the reduction in the incidence of postoperative delirium must be at least 5%.

In the following text, we described the many interventions that can be used directly by nursing staff to reduce the symptoms of delirium (Table 3). Finally, we indicated the most effective pharmacological treatments for delirium (Table 4) as well as pharmacological treatments that are ineffective (Table 5). All items in the three tables were ordered according to the level of evidence and quality of the studies from which they were extracted (John Hopkins Nursing Evidence-Based Practice [66]).

#### 3.2.1. Nursing Interventions

Nursing interventions that have shown greater levels of efficacy in the research studies included in this study are described below.

- Physiological: Maintain good nutrition and hydration statuses, manage pain adequately, and remove catheters promptly.
- Cognitive: Place clocks and calendars in the patient’s room to aid with temporal reorientation, general reorientation, and cognitive stimulation.
- Behavioural: Reduce preoperative anxiety, give psychosocial support, show concern and empathy, and listen attentively.
- Sensory: Maintain a good level of illumination, facilitate the use of hearing aids at an early stage, facilitate the use of glasses, and avoid excessive noise.
- Sleep and environment: Establish a daily routine in order to prevent disruption of the sleep–wake cycle, avoid the administration of medication or the taking of vital signs during the night where possible, and adjust routines in order to ensure uninterrupted sleep.
- Family involvement: Involve the patient’s family, avoid changes in personnel, and allow family to be present at meal-times.
- Patient safety and skin integrity: Avoid mechanical restraints where possible, and promote early mobility, walking, or directed exercise at least three times daily

#### 3.2.2. Effective Pharmacological Treatments

The drugs that seem to be more effective for reducing postoperative delirium are Haloperidol, Dexmedetomidine, Olanzapine, Quetiapine, Risperidone, and Melatonin

#### 3.2.3. Ineffective Pharmacological Treatments

Benzodiazepines, GABA agonists, Glutamate blockers, Haloperidol, Membrane stabilizers, and Donepezil have shown lower levels of treatment effectiveness.

## 4. Discussion

This integrative review attempted to conceptualize postoperative delirium and the conditions in which it appears to expand the body of knowledge about the factors that influence its appearance and development as well as the therapeutic measures that reduce the severity of the pathology.

As was mentioned, the aetiology of postoperative delirium is unknown [21,22,52,68,69,70,71], but it has been shown to be related to a large number of factors.

The reported incidence of postoperative delirium in the reviewed literature ranges from 10% to 90% [1,21,24,26,28].

Advanced age appears to be the principal risk factor predisposing a patient to the development of postoperative delirium. This was explored in a study published by Carrera Castro [14], which attributed this surgical risk factor to the deterioration in biological functions and functional capacity associated with aging.

De las Pozas Abril [1] established age as a factor related to the appearance of postoperative delirium, but it was not reported as an independent factor. In contrast, studies presented by Huang et al. [73], Veiga et al. [102], Ying et al. [17], Guo et al. [51], Van Der Sluis et al. [46], and Wang et al. [103] did establish age as an independent factor in the development of postoperative delirium. The results obtained in this review agree with the results of the study published by Bettelli and Neuner, showing that postoperative delirium occurs more frequently in elderly patients. This seems to coincide with the fact that age is a key factor in its development, in addition to the fact that with increasing age, the likelihood of suffering from other alterations that predispose a patient to the development of postoperative delirium is greater [35].

Regarding predisposing factors, the data obtained in this study agree with those obtained in a systematic review carried out by Aitken et al. [104], which suggested that age and previous cognitive decline are key factors in the development of delirium. Previous studies also agree that the occurrence of postoperative delirium can prolong the length of hospital stay [24].

Another factor closely related to the syndrome is postoperative pain. Álvarez-Bastidas et al. [105] found a two-fold increase in the risk of developing delirium in patients reporting medium–severe pain on the Visual Analogue Scale (VAS). Mei et al. [74] showed an association between pain and postoperative delirium in patients undergoing abdominal surgery requiring a general anaesthetic. The American Geriatrics Society [75] recommends that health professionals optimize post-operative pain control in older patients in order to prevent the occurrence of delirium. If possible, this should be conducted using non-opioid analgesics.

Cognitive deterioration and being male are other factors with strong relationships with the occurrence of postoperative delirium. Che-Sheng et al. [23] attributed the higher incidence of the syndrome among males compared with females to the fact that men have higher rates of auditory loss, comorbidities, and emergency hospital admissions.

It is necessary to develop new lines of investigation to establish the physiopathology of this disease and to drive the implementation of preventative measures and directed, scientifically proven treatments [76].

The identification of delirium continues to be a daily challenge in clinical practice. Diagnostic methods need to be improved and, for this, the involvement and training of nursing personnel is key [64].

In their guidelines, published in 2017, The European Society of Anaesthesiology [24] established that there are two highly sensitive scales that can be used to identify and diagnose postoperative delirium: the Nu-DESC and the CAM scales. However, it warns that, if the CAM scale is administered by untrained staff, it is far less accurate. They state that further investigation is necessary to identify the optimal tools for the identification of delirium on recovery wards.

Nursing interventions are principally focused on the prevention of delirium and the reduction in its symptoms after anaesthetic episodes.

Duarte et al. [65] established that nurses are taking on an ever more important role in the prevention, diagnosis, and treatment of delirium, since they are generally the first professionals to identify it. They recommend the implementation of measures and standard protocols within daily clinical practice. 

Among the non-pharmacological interventions, most frequently mentioned for the management of postoperative delirium is the reorientation of patients using objects such as clocks and calendars in the room, the maintenance of good nutrition and hydration practices, adequate management of pain, the maintenance of good illumination, the avoidance of excessive noise, facilitation of the use of hearing aids and glasses, and the promotion of early mobility.

A systematic review completed by Artuz et al. [6] analysed 30 studies concerning the nursing care of old adult patients with postoperative delirium and suggested that non-pharmacological measures are more important than pharmacological treatments for managing this condition. A study by Ocádiz-Carrasco et al. [22] supports this result, confirming that, currently, implementing non-pharmacological measures through a multidisciplinary approach is a strategy that is feasible, economic, and more effective than pharmacological treatments.

With respect to the pharmacological management of delirium, the most widely used drugs, according to the studies consulted, appear to be Haloperidol and Dexmedetomidine. However, it must be noted that there is no drug that is capable of totally eliminating the symptoms of postoperative delirium [8,9,11,20,21,24,27,34,35,36,38,39,42,53,58,60,63,76,77,78,79,106,107,108,109].

Various studies have shown that dexmedetomidine is an effective treatment for postoperative delirium [15,16,112], although without clear evidence [72,82,109]. Furthermore, another study suggested that dexmedetomidine is not an effective treatment for postoperative delirium [8], so we cannot conclude that its use is beneficial for the treatment of patients.

Several authors have proposed [11,53,106,110] the use of Haloperidol prophylactically in order to reduce the severity and duration of symptoms. However, due to the inconsistency of results and lack of evidence, the European Society of Anaesthesiology [24] guidelines do not advise its routine use, despite stating that there is evidence that Haloperidol, when used at low doses and as a preventative treatment, does reduce the incidence, severity, and duration of delirium.

Some of the studies reviewed suggest that the use of Benzodiazepines should be reserved for patients who are habitual users of this drug or who have a history of alcohol abuse, given that this category of drugs is, in fact, associated with the appearance of postoperative delirium [20,24,31,36,41,75,111].

Reviews completed by Borozdina et al. [80] and Popp et al. [60] suggest that melatonin should be administered prior to surgery in order to reduce the risk of the patient developing postoperative delirium. On the other hand, Guenther et al. [31] found that melatonin does not reduce the incidence of postoperative delirium in old adult patients who have undergone surgery. Other authors argue that the results of previous studies are too inconsistent to allow any recommendations to be determined regarding its preoperative use [24,72].

The studies included in this integrative review vary in terms of their level of methodological quality, as defined in the John Hopkins Nursing Evidence-Based Practice [66].

The heterogeneity of the studies and the results found highlight the need for more randomized clinical studies to be performed in this area. This would allow us to obtain a better understanding, based on scientific evidence, on postoperative delirium and give us new data that may help to uncover the unknown factors surrounding this condition.

### 4.1. Study Limitations

One of the principal limitations of this study is that, despite the high number of articles reviewed, they were very heterogeneous in nature, and there was a paucity of randomized clinical trials and systematic reviews. Furthermore, the similarity between the terms “postoperative delirium” and “emergency delirium” may have made it difficult to specifically search for articles on this topic.

### 4.2. Clinical Implications

The aetiology of postoperative delirium is not clear. Due to its high incidence and its clinical and economic repercussions, it is essential to train healthcare staff (especially nurses as staff members who are in close contact with the patient) in the recognition and management of postoperative delirium. As a future line of research, we suggest conducting a greater number of randomized clinical trials on the use of nursing interventions in the treatment of postoperative delirium.

## 5. Conclusions

The aetiology of postoperative delirium is still unknown, and no treatments exist to eliminate this pathology. Despite there being several different theories concerning the physiopathology of the condition, its causal mechanism is unclear, and there are no specific treatments that appear to efficiently counteract it.

Advanced age appears to be the principal risk factor for developing postoperative delirium, while pain and prior cognitive deterioration seem to be closely related to the development of this pathology. Interventions such as reorientation, pain management, and ensuring the patient receives good nutrition and hydration, in addition to the use of Haloperidol and Dexmedetomidine, seem to decrease the severity of delirium.

The role of nursing staff is fundamental in the prevention, diagnosis, and management of this pathology, given that these professionals are usually the first to notice symptoms.

## Figures and Tables

**Table 1:** Predisposing factors.

Predisposing Factors		
Advanced age [1,2,3,4,8,14,16,17,18,19,24,26,30,31,32,38,40,42,44,45,47,49,50,51,53,54,58,59,61,67,68,69,70,71,72,73,74,75,76,77,78,79,80,81,82,83,84,85,86,87,88,89,90,91,92,93,94,95,96,97,98,99,100,101,102,103,104,105,106,107,108,109] Pre-existing cognitive deterioration [1,3,8,9,14,16,18,31,32,40,45,46,54,60,67,76,78,79,80,81,91,94,97,110,111,112,113,114,115,116] Pain [2,4,14,30,31,32,34,69,70,71,75,79,80,91,100,104,111,117,118,119,120,121,122] Elevated ASA score [14,19,31,38,40,42,48,49,75,84,88,118] Alcohol abuse [16,23,30,32,44,67,81,90,91,97,101,121] Hypoxemia [23,30,67,70,76,80,91] Male sex [14,18,32,39,49,51,58,87,88,101,103,122] Metabolic disturbances, Water–electrolyte imbalance [16,30,31,75,76,80,81,94] Vision impairment [8,23,32,54,78,89,97] Auditory impairment [3,32,54,78,83,89,97] Disrupted sleep patterns [2,9,16,32,70,75,76,81,91] Depression [3,32,70,78,80,89,90,91,97] Previous episodes of delirium [14,32,80,81,88,95,97] Dehydration [2,16,32,76,80,81,91] Cerebrovascular disease [26,51,57,80,81,100] Intraoperative hypotension [19,59,75,80,120]	Sepsis [16,75,76,80,81] Reduced cerebral SaO2 [14,81,90,101] Renal insufficiency [32,75,81,84,91] Intraoperative haemorrhage [19,31,32,74] Low mini-mental state score [18,29,39,56,62] Dementia [3,58,70,78,97] Diabetes Mellitus type [19,29,40,44,80,88] Low level of education [4,9,28,58,67] Hypertension [75,80,87,88] Hypotension [14,16,23,76,81] High creatinine level [29,84,100] Immobilisation [30,32,75,91] Anaemia [44,70,81,83,91] Arterial fibrillation [17,26,44,83,90] Abnormally high albumin level [3,44,60,74,100] Cerebral aging [16,81,95] Functional deficit [29,78,89,91] Malnutrition [2,3,19,91] Sensory impairments [8,70,78] Parkinson’s disease [57,95] Smoking [38,44,83,87,90,101] Haematocrit < 30% [57,81] High PCR [29,84,100] Functional dependence [32,70] Drug use [80,97] Endocrine disorders [16,94] Urinary tract infections [81,95]	Hepatic insufficiency [32,113] Respiratory infection [81,95] Hyperglycaemia [23,100] Poor nutrition [80,81] Elevated APACHE II score [25] Low Barthel index [21] Low BMI [18] Low pre-op NEECAM score [39] High Bilirubin level [101] High postoperative level of Lactic acid [83] Social isolation [95] Myocardial ischemia [81,108] Neurotransmitter/receptor dysfunction [94] Preoperative anxiety [104] Lack of familiar environment [95] Emotional stress [32] Infection of the surgical wound [81] Shock [80] Cardiopulmonary disorder [108] Gastrointestinal disorder [81] Sleep apnoea [38] Hypercapnia [76] Hyperthermia [75] Thrombocytopenia [81] COPD [81] Hypothyroidism [80] HIV [32]

The numbers in the table refer to the reference numbers indicated in Table 6.

**Table 2:** Precipitating factors.

Precipitating Factors		
Benzodiazepines [15,16,19,30,32,40,44,49,56,58,70,76,79,83,87,89,91,101,104] Cardiac surgery [32,55,70,75,76,91] Heart-lung bypass [17,84,113,116] Duration of surgery [9,16,24,28,31,44,67,79,83,100,101,103] Emergency surgery [24,55,70,75,80,96] Orthopaedic surgery [28,32,39,55,57,70,76,81,88,98] Type of surgery [18,49,67,83,95,103,104] Major transfusion [17,18,19,38,47,49,60,75,100] Admission to UCI [16,25,32,47,55,57,76,82,118] Psychotropics [46,56,58,78,90] Inhalational anaesthetic [49,65,94,101,103]	Opioids [12,49,69,75,83] Anticholinergics [32,73,76,79,81,117] Foley catheterization or other invasive procedures [16,30,32,76,91,101] General anaesthetic [58,94,119] Duration of anaesthesia [58,67,75] Loneliness [4,14,47] Analgesics [58,87] Pharmacological suppression, physical restriction [32,70] Mechanical ventilation [2,79] Duration of orotracheal intubation [17,26] Institutionalisation [78,114]	Poor family support [16,76] Fresh frozen plasma [49,96] Length of stay [14] Abdominal surgery [75] Neurovascular surgery [75] Polypharmacy [45] Balloon counter-pulsation [80] delay in emergence from anaesthesia [25] Hospitalisation [30] Urological surgery [28] GABA [73] Adrenaline [26]

The numbers in the table refer to the reference numbers indicated in Table 6.

**Table 3:** Nursing interventions to reduce postoperative delirium.

Nursing Interventions	
**Physiological** Maintain good nutrition and hydration [3,9,16,33,44,69,81,83,94,119] Manage pain adequately [7,9,10,33,34,55,59,69,82] Remove catheters promptly [3,7,9,55,79,82] Ensure adequate oxygenation [2] Manage bladder and bowel care [94] Minimize the risk of aspiration pneumonia [94] Avoid prolonged hypotension [107] **Cognitive** Place clocks and calendars in the patient’s room to aid in temporal reorientation [3,7,8,13,55,62,76,81,94,119] General reorientation [55,64,82,119] Cognitive stimulation [7,44,76,81,94,119] Read letters or books to the patient or show the patient family photographs [13,55] Implement strategies to help the patient differentiate between day and night by, for example, showing them pictures of the sun or moon [7,55] Use music [7,55] Give the patient access to objects that will help them orient themselves [119] Encourage reading, watching TV, and listening to music [7,55] Place objects such as photographs where they are easily visible to the patient [55] Avoid excessive perioperative and postoperative sedation [2] **Behavioural** Reduce preoperative anxiety [107] Give psychosocial support [16] Show concern and empathy; listen attentively [55] Do not dismiss concerns expressed by the patient [107] Offer realistic solutions and avoid threats [55] Use open questions and try to discover the source of patient concerns Be assertive; use sentences that are short and clear [107] Use acupuncture and acupressure (at the “Shenmen” and “Point Zero” auricular acupuncture points) [107]	**Sensory** Maintain a good level of illumination [7,13,55,62,70,76,79,94,119] Facilitate the use of hearing aids at an early stage [3,7,33,55,62,69,76,79,102,119] Facilitate the use of glasses [3,7,11,13,62,69,79,102] Avoid excessive noise [3,7,33,55,81,95,119] Facilitate the use of dentures [10,13,50] Visual and auditory stimulation [76,81] Avoid artificial lighting [81,82] **Sleep and Environment** Establish a daily routine in order to prevent disruption of the sleep–wake cycle [13,81,95] Where possible, avoid the administration of medication or the taking of vital signs during the night [55,64,81] Adjust routines in order to ensure uninterrupted sleep [119] Back massage [46,119] Enable the patient to obtain adequate rest [11,35] Give the patient peace and quiet and maintain a pleasant environment (without unwanted noise or visits) [55,95] Enquire as to whether the patient was already taking medication to aid sleep [119] Allow a warm milky drink to be taken before sleep [46] Provide warm drinks for the patient [119] **Family involvement** Involve the patient’s family [10,55,62,64,69] Avoid changes in personnel [13,62,94] Allow the family to be present at meal-times [7,55] It may be necessary to require a family member or someone who is close to accompany the elderly patient [95] Use carers’ names [119] **Patient Safety and Skin Integrity** Where possible, avoid mechanical restraints [2,16,55,64,79,81,95,119] Promote early mobility [3,7,10,33,34,44,55,59,69,76,82,95] Walking or directed exercise at least three times daily [7,55,76,119] Protect the patient from falls [94] Care of pressure points [2,94]

The numbers in the table refer to the reference numbers indicated in Table 6.

**Table 4:** Effective pharmacological methods for the treatment of postoperative delirium.

Effective Pharmacological Treatments		
Haloperidol [8,9,11,15,16,33,47,54,64,70,74,78,81,89,92,94,97,102] Dexmedetomidine [8,10,11,15,16,33,37,47,54,64,70,78,81,89,94,97,102] Benzodiazepines [3,10,87,89,91] Olanzapine [9,10,11,15,47,75,82,97,115] Quetiapine [11,47,64,70,74,75,78,81,97,115] Risperidone [9,11,43,47,64,75,81,82,89,97] Clonidine [3,107]	Chlorpromazine [87,115] Propofol [107] Melatonin [9,70,78] Hydroxyzine + Midazolam [107] Acetaminophen [117] Antipsychotics [82] Aprotinin [94,112] Peripheral nerve blocks [119]	Ziprasidone [10,82] Statins [79,97] Fentanyl [107] Ketamine [33] Levomepromazine [87] Nefopam [101] Rivastigmine [97] Sufentanil [107]

The numbers in the table refer to the reference numbers indicated in Table 6.

**Table 5:** Ineffective pharmacological methods for the treatment of postoperative delirium.

Ineffective Pharmacological Treatments		
Benzodiazepines [2,9,10,15,44,69,91] Melatonin [2,42,79] GABA agonists [94,112] Glutamate blockers [95,112] Haloperidol [2,64] Membrane stabilizers [95,112] Donepezil [78,79]	Dexmedetomidine [28] NMDA agonists [106] Antioxidants [15] Barbiturates [41] Ca++ or Na+ blockers [112] Gabapentin [66] Ganglioside GM1 [112]	Leukocyte adhesion inhibitors or growth factor [95] Ketamine [7] Nimodipine [2] Remacemide [112] Rivastigmine [79] Tryptophan [111]

The numbers in the table refer to the reference numbers indicated in Table 6.

**Table 6:** Levels of evidence and quality of the included studies.

Number	Authors	Year	Evidence and Quality	Number	Authors	Year	Evidence and Quality
1	Evered et al.	2018	I A	61	An et al.	2018	II A
2	De las Pozas Abril et al.	2011	I A	62	Chung-Sik et al.	2016	III A
3	Honda et al.	2018	III A	63	Rodríguez Soto et al.	2015	V C
4	Styra et al.	2018	III A	64	Alcoba Pérez et al.	2014	III A
5	Shawna Greiner et al.	2019	I A	65	Duarte Martinez et al.	2018	V A
6	Artuz Diaz et al.	2016	II A	66	Dearholt et al.	2012	I A
7	Card et al.	2014	III A	67	Ewan et al.	2010	I A
8	Pavone et al.	2018	III A	68	Koster et al.	2010	I A
9	Wang et al.	2018	III A	69	Vásquez-Marquez et al.	2011	V B
10	Sosa Morales et al.	2017	III A	70	Steiner et al.	2011	V A
11	Rodríguez et al.	2017	III A	71	Renger et al.	2018	III B
12	Miller et al.	2018	I A	72	Gräsner et al.	2016	I A
13	Punjasawadwong et al.	2018	I A	73	Huang et al.	2019	I A
14	Carrera Castro et al.	2014	IV A	74	Mei et al.	2016	III B
15	Dotti et al.	2017	III B	75	The American Geriatrics Society.	2015	II A
16	Van Grootven et al.	2015	III B	76	Vásquez-Marquez et al.	2012	V B
17	Guo et al.	2016	II A	77	Jia et al.	2014	I A
18	Fields et al.	2018	III A	78	Mosk et al.	2018	III A
19	Hesse et al.	2018	III A	79	Read et al.	2017	V C
20	Calderón Delgado et al.	2017	III A	80	Borozdina et al.	2018	I B
21	Peralta-Zamora et al.	2012	V B	81	Luo et al.	2018	II A
22	Ocádiz-Carrasco	2013	III A	82	Jee et al.	2017	I A
23	Chu et al.	2015	II A	83	Munk et al.	2013	III A
24	Aldecoa et al.	2017	IV A	84	Goins et al.	2018	III B
25	Riegger et al.	2018	I B	85	Kassie et al.	2017	I A
26	Fritz et al.	2018	III A	86	Soto Martín	2015	III B
27	Mimi et al.	2018	I A	87	Li et al.	2017	I A
28	MacKenzie et al.	2018	I A	88	González Masís et al.	2014	III A
29	Espinosa Calderón et al.	2017	III B	89	Kratz et al.	2015	III A
30	Lira et al.	2018	V B	90	Smulter et al.	2017	III A
31	Guenther et al.	2011	III A	91	Lee et al.	2018	III A
32	Guenther et al.	2016	III A	92	Chan et al.	2018	III A
33	Sanson et al.	2018	III A	93	Shin et al.	2016	I A
34	Mcdaniel et al.	2012	V B	94	Sánchez et al.	2019	I A
35	Bettelli et al.	2017	V B	95	Munk et al.	2016	I A
36	Celis et al.	2017	V A	96	Esteve et al.	2013	V A
37	Li et a.	2017	III A	97	Smulter et al.	2019	III B
38	Lee et al.	2013	III B	98	Lee et al.	2019	I A
39	Fok et al.	2015	I A	99	Carranza Salas et al.	2017	III A
40	Dong et al.	2017	III A	100	Wen et al.	2018	II A
41	Marcantonio et al.	2017	V A	101	Subramaniam et al.	2019	I A
42	Nuñez Ureña et al.	2017	III B	102	Veiga et al.	2012	III A
43	Hempenius et al.	2014	III A	103	Wang et al.	2018	I A
44	Dan et al.	2017	I B	104	Aitken et al.	2017	II A
45	Cheol et al.	2018	I A	105	Alvarez-bastidas et al.	2018	III C
46	Nadler et al.	2017	I B	106	Nazemi et al.	2017	I B
47	Van Der Sluis et al.	2016	IIA	107	Ha et al.	2018	III A
48	Kang et al.	2019	III A	108	Calderón Rodríguez et al.	2018	V B
49	Xing et al.	2016	III A	109	Steiner et al.	2012	V A
50	Deiner et al.	2017	I A	110	Fukata et al.	2016	II A
51	Chevillon et al.	2015	I A	111	Rincon Franco et al.	2017	I A
52	Guo et al.	2016	IIA	112	Shankar et al.	2018	I A
53	Chacón Zamora et al.	2014	V C	113	Koskderelioglu et al.	2017	III A
54	Romero Luna et al.	2014	III B	114	Winter et al.	2015	III A
55	Jimenez Ardila et al.	2013	IV A	115	J. Smith et al.	2016	III C
56	Van Meenen et al.	2014	II A	116	Hernández et al.	2012	III C
57	Ogawa et al.	2017	III A	117	Järvelä et al.	2017	III B
58	Duan et al.	2018	I A	118	Vilchis-rentería et al.	2012	V B
59	Langer et al.	2019	I A	119	Stephani Hernández et al.	2014	III A
60	Popp et al.	2012	V A	120	Kassie et al.	2018	II A

## Data Availability

Data available in publicly accessible repositories.

## References

[1] Evered L., Silbert B., Knopman D.S., Scott D.A., Dekosky S.T., Rasmussen L.S. (2018). Recommendations for the Nomenclature of Cognitive Change Associated with Anaesthesia and Surgery—2018. Anesthesiology.

[2] de las Pozas Abril J. (2011). Postoperative Delirium and factors related in a Unit Care of Cardiac Surgery. Nure Investig..

[3] Honda S., Furukawa K., Nishiwaki N., Fujiya K., Omori H., Kaji S., Makuuchi R., Irino T., Tanizawa Y., Bando E. (2018). Risk factors for Postoperative Delirium after Gastrectomy in gastric cancer patients. World J. Surg..

[4] Styra R., Larsen E., Dimas M.A., Baston D., Elgie-Watson J., Flockhart L., Lindsay T.F. (2018). The effect of preoperative cognitive impairment and type of vascular surgery procedure on postoperative delirium with associated cost implications. J. Vasc. Surg..

[5] Shawna Greiner C., Kremer M.J. (2019). Clarifying the Confusion of Adult Emergence Delirium. AANA J..

[6] Artuz Diaz D.E., Burgos Chaverra E.L., Garcia Sanchez E.M., Gonzalez Urueta K.D., Ortega Dehorta K.D. (2016). Cuidados de Enfermería a Adultos Mayores Con Delirium Postquirúrgico.

[7] Card E., Pandharipande P., Tomes C., Lee C., Wood J., Nelson D., Graves A., Shintani A., Ely E., Hughes C. (2014). Emergence from general anaesthesia and evolution of delirium signs in the post anaesthesia care unit. Br. J. Anaesth..

[8] Pavone K.J., Cacchione P.Z., Polomano R.C., Winner L., Compton P. (2018). Evaluating the use of Dexmedetomidine for the reduction of delirium: An integrative review. Hear Lung.

[9] Wang Y., Shen X. (2018). Postoperative delirium in the elderly: The potential neuropathogenesis. Aging Clin. Exp. Res..

[10] Sosa Morales G., Alonso Cabrera E., Martínez Oquendo A., Montes de Oca Montano J.L., León Valdivies Y.J. (2017). El Delirio En Ancianos Hospitalizados. Un Estudio en la Unidad de Cuidados Intermedios Quirúrgicos de Cienfuegos. Cuba.

[11] Rodríguez J. (2017). Delirium Perioperatorio. Rev. Méd. Clín. Condes.

[12] Miller D., Lewis S., Pritchard M., Schofield-robinson O., Shelton C., Alderson P., Smith A.F. (2018). Intravenous versus inhalational maintenance of anaesthesia for postoperative cognitive outcomes in elderly people undergoing non-cardiac surgery. Cochrane Database Syst. Rev..

[13] Punjasawadwong Y., Chau-In W., Laopaiboon M., Punjasawadwong S., Pin-On P. (2018). Processed electroencephalogram and evoked potential techniques for amelioration of postoperative delirium and cognitive dysfunction following non-cardiac and non- neurosurgical procedures in adults. Cochrane Database Syst. Rev..

[14] Carrera Castro C. (2014). Delirium postoperatorio en cirugía general, el fantasma de nuestros abuelos. Enferm. Glob..

[15] Dotti S., Montes De Oca O., Bigalli D., Gutierrez F., Russo N., Pouso M. (2017). Análisis prospectivo sobre incidencia acumulada de delirio en el posoperatorio de cirugía cardíaca. Rev. Urug. Cardiol..

[16] Van Grootven B., Detroyer E., Devriendt E., Sermon A., Deschodt M., Flamaing J., Dubois C., Milisen K. (2015). Is preoperative state anxiety a risk factor for postoperative delirium among elderly hip fracture patients?. Geriatr. Gerontol. Int..

[17] Guo Y., Fan Y. (2016). A Preoperative, Nurse-Led Intervention Program Reduces Acute Postoperative Delirium. Am. Assoc. Neurosci. Nurse.

[18] Fields A., Huang J., Schroeder D., Sprung J., Weingarten T. (2018). Agitation in adults in the post-anaesthesia care unit after general anaesthesia. Br. J. Anaesth..

[19] Hesse S., Kreuzer M., Hight D., Gaskell A., Devari P., Singh D., Taylor N.B., Whalin M.K., Lee S., Sleigh J.W. (2018). Association of electroencephalogram trajectories during emergence from anaesthesia with delirium in the post-anaesthesia care unit: An early sign of postoperative complications. Br. J. Anaesth..

[20] Calderón Delgado T.B., Jara Alvarado J.R. (2017). Disfunción Cognitiva Postoperatoria en Pacientes Mayores de 60 Años de Edad Sometidos a Procedimientos de Anestesia General Mediante la Valoración del Test de MOCA en el Hospital San Francisco de Quito Durante el Periodo de Junio-Agosto Del Año 2016.

[21] Peralta-Zamora E. (2012). Estrategias para disminuir la agitación y el delirio postoperatorio en anestesia ambulatoria. Rev. Mex. Anestesiol..

[22] Ocádiz-Carrasco J., Gutiérrez-Padilla R.A., Páramo-Rivas F., Tovar-Serrano A., Hernández-Ortega J.L. (2013). Programa preventivo del delirio postoperatorio en ancianos. Cir. Cir..

[23] Chu C.-S., Liang C.-K., Chou M.-Y., Lin Y.-T., Hsu C.-J., Chou P.-H., Chu C.-L. (2015). Short-Form Mini Nutritional Assessment as a useful method of predicting the development of postoperative delirium in elderly patients undergoing orthopedic surgery. Gen. Hosp. Psychiatry.

[24] Aldecoa C., Bettelli G., Bilotta F., Sanders R.D., Audisio R., Borozdina A., Cherubini A., Jones C., Kehlet H., MacLullich A. (2017). European Society of Anaesthesiology evidence-based and consensus-based guideline on postoperative delirium. Eur. J. Anaesthesiol..

[25] Riegger H., Hollinger A., Seifert B., Toft K., Blum A., Zehnder T., Siegemund M. (2018). Baden Prevention and Reduction of Incidence of Postoperative Delirium Trial (PRIDe): A phase IV multicenter, blind clinical trial of ketamine versus haloperidol for prevention of postoperative delirium. BMC.

[26] Fritz B.A., Maybrier H.R., Avidan M.S. (2018). Intraoperative electroencephalogram suppression at lower volatile anaesthetic concentrations predicts postoperative delirium occurring in the intensive care unit. Br. J. Anaesth..

[27] Mimi W., Yongxin L., Zhao D., Shiduan W. (2018). Perioperative dexmedetomidine reduces delirium after cardiac surgery: A meta-analysis of randomized controlled trials. J. Clin. Anesth..

[28] MacKenzie K.K., Britt-Spells A.M., Sands L.P., Leung J.M. (2018). Processed Electroencephalogram Monitoring and Postoperative Delirium. Anesthesiology.

[29] Espinosa Calderón H.P., Sosa Julia S., Mantilla Pinto X.R. (2017). Delirio Posoperatorio En Pacientes Geriátricos Sometidos a Anestesia General versus Neuroaxial, Medido por el Método de Evaluación de la Confusión, en el Hospital Eugenio Espejo y Hospital de la Policía Nacional, Agosto y Septiembre 2016.

[30] Lira D., Mar-Meza M., Montesinos R., Herrera-Pérez E., Cuenca J., Castro-Suárez S., Custodio N. (2018). Una complicación quirúrgica escasamente sospechada: La Disfunción Cognitiva Postoperatoria. Rev. Neuropsiquiatr..

[31] Guenther U., Radtke F.M. (2011). Delirium in the postanaesthesia period. Curr. Opin. Anesthesiol..

[32] Guenther U., Riedel L., Radtke F.M. (2016). Patients prone for postoperative delirium: Preoperative assessment, perioperative prophylaxis, postoperative treatment. Curr. Opin. Anesthesiol..

[33] Sanson G., Khlopenyuk Y., Milocco S., Sartori M., Dreas L., Fabiani A. (2018). Delirium after cardiac surgery. Incidence, phenotypes, predisposing and precipitating risk factors, and effects. Hear Lung.

[34] Mcdaniel M., Brudney C. (2012). Postoperative delirium: Etiology and management. Curr. Opin. Crit. Care.

[35] Bettelli G., Neuner B. (2017). Postoperative delirium: A preventable complication in the elderly surgical patient. Monaldi Arch. Chest Dis..

[36] Celis E., Vega Salazar F., Torres Marrugo V. (2017). Revisión comparativa de las guías de sedación, analgesia y delirio en pacientes críticos. Acta Colomb. Cuid. Intensiv..

[37] Li X., Yang J., Nie X.L., Zhang Y., Li X.Y., Li L.H., Wang D.X., Ma D. (2017). Impact of dexmedetomidine on the incidence of delirium in elderly patients after cardiac surgery: A randomized controlled trial. PLoS ONE.

[38] Lee J., Jung J., Jai Sung N., Yoo S., You Sun H. (2013). Perioperative Psycho-Educational intervention can reduce postoperative delirium in patients after Cardiac Surgery: A Pilot Study. Psychiatry Med..

[39] Fok M.C., Sepehry A.A., Frisch L., Sztramko R., Van Der Burg B.L.S.B., Vochteloo A.J.H., Chan P. (2015). Do antipsychotics prevent postoperative delirium? A systematic review and meta-analysis. Int. J. Geriatr. Psychriaty.

[40] Dong X., Hailin X., Huiyu T., Guozhu X. (2017). Preoperative C-Reactive Protein as a risk factor for Postoperative Delirium in elderly patients undergoing Laparoscopic Surgery for Colon Carcinoma. Biomed. Res. Int..

[41] Marcantonio A.J., Pace M., Brabeck D., Trzaskos A., Anderson R. (2017). Team Approach: Management of Postoperative Delirium in the elderly patient with femoral-neck fracture. JBJS Rev..

[42] Nuñez Ureña J.M., López Carrillo L., Hernández Luna A., Hardy Pérez A.E., Jaimes García J., Domínguez Cadena A., Vasquez Ceron J.A. (2017). Factores de Riesgo de Delirium Postoperatorio en la Unidad de Cuidados Intensivos.

[43] Hempenius L., Slaets J.P.J., Asselt D.Z.B., Van Schukking J., De Bock G.H., Wiggers T., van Leeuwen B.L. (2014). Interventions to prevent postoperative delirium in elderly cancer patients should be targeted at those undergoing nonsuperficial surgery with special attention to the cognitive impaired patients. Eur. J. Surg. Oncol..

[44] Dan L., Jie L., Huiying Z., Youzhong A. (2017). The influence of analgesic-based sedation protocols on delirium and outcomes in critically ill patients: A randomized controlled trial. PLoS ONE.

[45] Cheol L., Cheol Hyeong L., Gilho L., Jongmyeong L., Jihyo H. (2018). The effect of the timing and dose of dexmedetomidine on postoperative delirium in elderly patients after laparoscopic major non-cardiac surgery: A double blind randomized controlled study. J. Clin. Anesth..

[46] Nadler J.W., Evans J.L., Fang E., Preud’Homme X.A., Daughtry R.L., Chapman J.B., Bolognesi M.P., Attarian D.E., Wellman S.S., Krystal A.D. (2017). A randomised trial of peri operative positive airway pressure for postoperative delirium in patients at risk for obstructive sleep apnoea after regional anaesthesia with sedation or general anaesthesia for joint arthroplasty. Anesthesia.

[47] Van Der Sluis F.J., Buisman P.L., Meerdink M., Wouter B., van Etten B., de Bock G.H., van Leeuwen B.L., Pol R.A. (2016). Risk factors for postoperative delirium after colorectal operation. Surgery.

[48] Kang S.Y., Sang Won S., Joo Yong K. (2019). Comprehensive risk factor evaluation of postoperative delirium following major surgery: Clinical data warehouse analysis. Neurol. Sci..

[49] Xin J., Dong C., Yahao L., Zhongshi L. (2016). Risk factors for postoperative delirium after spine surgery in middle- and old-aged patients. Aging Clin. Exp. Res..

[50] Deiner S., Luo X., Lin H.-M., Sessler D.I., Saager L., Sieber F.E., Lee H.B., Sano M., Jankowski C., Bergese S. (2017). Intraoperative infusion of Dexmedetomidine for prevention of Postoperative Delirium and Cognitive Dysfunction in elderly patients undergoing major elective Noncardiac Surgery. JAMA Surg..

[51] Chevillon C., Hellyar M., Madani C., Kerr K., Kim S.C. (2015). Preoperative education on Postoperative Delirium, anxiety, and knowledge in Pulmonary Thromboendarterectomy patients. Am. J. Crit. Care..

[52] Guo Y., Jia P., Zhang J., Wang X., Jiang H., Jiang W. (2016). Prevalence and risk factors of postoperative delirium in elderly hip fracture patients. J. Int. Med. Res..

[53] Chacón Zamora M. (2014). Delirio postquirúrgico en fractura de cadera del paciente adulto mayor. Rev. Clín. Esc. Med..

[54] Romero Luna D.I., Cuitláhuac Márquez Z., González Hidalgo E. (2014). Frecuencia de la Disfunción Cognitiva Posoperatoria (DCPO) en Adultos, Sometidos a Colecistectomía Laparoscópica Programada Bajo Anestesia General Balanceada, En Pacientes Premedicados Con Midazolam.

[55] Jiménez Ardila Y.B., Marténez Castro J., Peña Aguirre Y.V. (2013). Guía De Enfermería Para La Prevención Y Manejo No Farmacológico del Delirium en Pacientes en Postoperatorio de Cirugía Cardiovascular en la Unidad Cardiovascular de la Fundación Cardioinfantil de Bogotá.

[56] Van Meenen L.C.C., Van Meenen D.M.P., De Rooij S.E., ter Riet G. (2014). Risk Prediction Models for Postoperative Delirium: A Systematic Review and Meta-Analysis. Aging Surg..

[57] Ogawa M., Izawa K.P., Satomi-Kobayashi S., Kitamura A., Tsuboi Y., Komaki K., Ono R., Sakai Y., Tanaka H., Okita Y. (2017). Preoperative exercise capacity is associated with the prevalence of postoperative delirium in elective cardiac surgery. Aging Clin. Exp. Res..

[58] Duan X., Coburn M., Rossaint R., Sanders R.D., Waesberghe J.V., Kowark A. (2018). Efficacy of perioperative dexmedetomidine on postoperative delirium: Systematic review and meta-analysis with trial sequential analysis of randomised controlled trials. Br. J. Anaesth..

[59] Langer T., Santini A., Zadek F., Chiodi M., Pugni P., Cordolcini V., Bonanomi B., Rosini F., Marcucci M., Valenza F. (2019). Intraoperative hypotension is not associated with postoperative cognitive dysfunction in elderly patients undergoing general anesthesia for surgery: Results of a randomized controlled pilot trial. J. Clin. Anesth..

[60] Popp J., Arlt S. (2012). Prevention and treatment options for postoperative delirium in the elderly. Curr. Opin. Psychiatry.

[61] An Y., Jin Y., Jin T., Hur E.Y., Lee S. (2018). Operative and Anesthetic Factors Influencing on Delirium in the Intensive Care Unit: An analysis of Electronic Health Records. J. Clin. Nurs..

[62] Chung-Sik O., Ka Young R., Tae-Gyoon Y., Nam-Sik W., Seung Wan H., Seong-Hyop K. (2016). Postoperative Delirium in Elderly Patients Undergoing Hip Fracture Surgery in the Sugammadex Era: A Retrospective Study. Biomed. Res. Int..

[63] Rodríguez Soto Y. (2013). Delirium Postoperatorio: Implicación clínica y manejo. Rev. Med. Costa Rica Centroam..

[64] Alcoba Pérez Á., Ciria Poza S., Carracedo Catalán C., García Fernández A., Marcos Vidal J.M. (2014). Valoración de la concordancia entre la escala CAM-ICU y la nursing delirium screening scale en el postoperatorio de cirugía cardiaca en una unidad de críticos. Enferm. Intensiv..

[65] Duarte Martínez D.M. (2018). Factores de Riesgo en Pacientes Adultos Para el Desarrollo de Delirium Una Perspectiva Desde el Cuidado de Enfermería.

[66] Dearholt S.L., Dang D. (2012). Evidence-Based Practice: Model and Guidelines Johns Hopkins Nursing.

[67] Ewan S., Noel-Storr A., William C. (2010). The Impact of General and Regional Anesthesia on the Incidence of Post- Operative Cognitive Dysfunction and Post-Operative Delirium: A Systematic Review with Meta-Analysis. J. Alzheimers Dis..

[68] Koster S., Hensens A.G., Schuurmans M.J., Van Der Palen J. (2010). Risk factors of delirium after cardiac surgery A systematic review. Eur. J. Cardiovasc. Nurs..

[69] Vásquez-márquez I., Castellanos-Olivares A. (2011). Alteraciones cognitivas y postoperatorias en el paciente geriátrico. Rev. Mex. Anestesiol..

[70] Steiner L.A. (2011). Postoperative delirium. Part 1: Pathophysiology and risk factors. Eur. J. Anesthesiol..

[71] Rengel K.F., Pandharipande P.P., Hughes C.G. (2018). Postoperative delirium. Presse Med..

[72] Gräsner J.-T., Lefering R., Koster R.W., Masterson S., Böttiger B.W., Herlitz J., Wnent J., Tjelmeland I.B., Ortiz F.R., Maurer H. (2016). EuReCa ONE 27 Nations, ONE Europe, ONE Registry. Resuscitation.

[73] Huang J., Qi H., Lv K., Chen X., Zhuang Y., Yang L. (2019). Emergence Delirium in Elderly Patients as a Potential Predictor of Subsequent Postoperative Delirium: A Descriptive Correlational Study. J. PeriAnesth. Nurs..

[74] Mei X., Tong J. (2016). The plasma levels of brain-derived neurotrophic factor are positively associated with emergence agitation in the elderly after gastrointestinal surgery. J. Anesth..

[75] Inouye S.K., Robinson T., Blaum C., Boustani M., Busby-Whitehead J., Chalian A. (2015). The American Geriatrics Society Expert Panel on Postoperative Delirium in Older Adults. Postoperative Delirium in Older Adults: Best Practice Statement from the American Geriatrics Society. J. Am. Coll. Surg..

[76] Vásquez Márquez I., Castellanos Olivares A. (2011). Delirio postoperatorio en el paciente geriátrico. Rev. Mex. Anestesiol..

[77] Jia Y., Jin G., Guo S., Gu B., Zujian J., Xing G., Li Z. (2014). Fast-track surgery decreases the incidence of postoperative delirium and other complications in elderly patients with colorectal carcinoma. Langenbeck’s Arch. Surg..

[78] Mosk C.A., van Vugt J.L., de Jonge H., Witjes C.D., Buettner S., Ijzermans J.N., van der Laan L. (2018). Low skeletal muscle mass as a risk factor for postoperative delirium in elderly patients undergoing colorectal cancer surgery. Clin. Interv. Aging.

[79] Read M.D., Maani C.V., Blackwell S. (2017). Dexmedetomidine as a Rescue Therapy for Emergence Delirium in Adults: A Case Series. Case Rep..

[80] Borozdina A., Qeva E., Cinicola M., Bilotta F. (2018). Perioperative cognitive evaluation. Curr. Opin. Anesthesiol..

[81] Luo C., Zou W. (2018). Cerebral monitoring of anaesthesia on reducing cognitive dysfunction and postoperative delirium: A systematic review. J. Int. Med. Res..

[82] Jee Y.S., You H.J., Sung T.Y., Cho C.K. (2017). Effects of nefopam on emergence agitation after general anesthesia for nasal surgery. Medicine.

[83] Munk L., Peter L., Andersen H., Gögenur I. (2013). Emergence delirium. Clin. Featur..

[84] Goins A.E., Smeltz A., Ramm C., Strassle P.D., Teeter E.G., Vavalle J.P., Kolarczyk L. (2018). General Anesthesia for transcatheter Aortic Valve replacement: Total Intravenous Anesthesia is associated with less Delirium as compared to volatile agent technique. J. Cardiothorac. Vasc. Anesth..

[85] Kassie G.M., Nguyen T.A., Ellett L.M.K., Pratt N.L., Roughead E.E. (2017). Preoperative medication use and postoperative delirium: A systematic review. BMC Geriatr..

[86] Soto Martin V., Ojeda González J.J., Dávila Cabo de Villa E. (2015). Síndrome confusional agudo posanestesia en el paciente geriátrico de urgencia. Rev. Cuba. Anestesiol. Reanim..

[87] Li T., Yeung J., Li J., Zhang Y., Melody T., Gao Y., Wang Y., Lian Q., Gao F. (2017). Comparison of regional with general anaesthesia on postoperative delirium (RAGA-delirium) in the older patients undergoing hip fracture surgery: Study protocol for a multicentre randomised controlled trial. BMJ Open.

[88] González Masis J.R., Cordero Escobar I., Rassi Llanes D., Mora Díaz I. (2014). Utilidad del Minimental State en el diagnóstico de disfunción cognitiva posoperatoria del anciano. Rev. Cuba. Anestesiol. Reanim..

[89] Kratz T., Heinrich M., Schlauß E., Diefenbacher A. (2015). Preventing Postoperative Delirium. Dtsch. Ärztebl. Int..

[90] Smulter N., Lingehall H.C., Yngve G., Olofsson B., Gunnar K., Appelblad M., Svenmarker S. (2017). Disturbances in oxygen balance during Cardiopulmonary Bypass: A risk factor for Postoperative Delirium. J. Cardiothorac. Vasc. Anesth..

[91] Lee D.S., Lee M.Y., Park C.M., Kim D.I., Kim Y.W., Park Y.J. (2018). Preoperative statins are associated with a reduced risk of postoperative delirium following vascular surgery. PLoS ONE.

[92] Chan B., Aneman A. (2018). A prospective, observational study of cerebrovascular autoregulation and its association with delirium following cardiac surgery. Anesthesia.

[93] Shin J.E., Kyeong S., Lee J.S., Park J.Y., Lee W.S., Kim J.J., Yang K.H. (2016). A personality trait contributes to the occurrence of postoperative delirium: A prospective study. BMC Psychiatry.

[94] Sánchez A., Thomas C., Deeken F., Wagner S., Klöppel S., Kentischer F. (2019). Quality of life: Reduction of delirium risk and postoperative cognitive dysfunction after elective procedures in older adults—study protocol for a stepped-wedge cluster randomized trial (PAWEL Study). Trials.

[95] Munk L., Andersen G., Møller A.M. (2016). Post-anaesthetic emergence delirium in adults: Incidence, predictors and consequences. Acta Anaesthesiol. Scand..

[96] Esteve N., Valdivia J., Ferrer A., Mora C., Ribera H., Garrido P. (2013). Influyen las técnicas anestésicas en los resultados postoperatorios? Parte, I. Rev. Esp. Anestesiol. Reanim..

[97] Smulter N., Lingehall H.C., Gustafson Y., Olofsson B., Engström G.K. (2019). The use of a screening scale improves the recognition of delirium in older patients after cardiac surgery–a retrospective observational study. J. Clin. Nurs..

[98] Lee S., Choi S.J., In C.B., Sung T. (2019). Effects of tramadol on emergence agitation after general anesthesia for nasal surgery. Medicine.

[99] Carranza Salas E. (2017). Revisión Crítica: Efectividad de la Intervención de Enfermería en el Preoperatorio Para Reducir el Delirio en Pacientes de Cirugía Mayor.

[100] Wen J., Zeng H., Li Z., He G., Jin Y. (2018). Pharmacologic interventions for preventing delirium in adult patients after cardiac surgery. Medicine.

[101] Subramaniam B., Shankar P., Shaefi S., Mueller A., O’Gara B., Banner-Goodspeed V., Gallagher J., Gasangwa D., Patxot M., Packiasabapathy S. (2019). Effect of intravenous Acetaminophen vs Placebo combined with Propofol or Dexmedetomidine on Postoperative Delirium among older patients following Cardiac Surgery. JAMA.

[102] Veiga D., Luis C., Parente D., Fernandes V., Botelho M., Santos P., Abelha F. (2012). Postoperative Delirium in Intensive Care Patients: Risk Factors and Outcome. Braz. J. Anesthesiol..

[103] Wang C.G., Qin Y.F., Wan X., Song L.C., Li Z.J., Li H. (2018). Incidence and risk factors of postoperative delirium in the elderly patients with hip fracture. J. Orthop. Surg. Res..

[104] Aitken S.J., Blyth F.M., Naganathan V. (2017). Incidence, prognostic factors and impact of postoperative delirium after major vascular surgery: A meta-analysis and systematic review. Vasc. Med..

[105] Alvarez-Bastidas L., Morales-Vera E., Valle-Leal J.G., Marroquín González J. (2018). Delirio en el adulto mayor sometido a anestesia: Factores asociados. Colomb. J. Anesthesiol..

[106] Nazemi A.K., Gowd A.K., Carmouche J.J., Kates S.L., Albert T.J., Behrend C.J. (2017). Prevention and management of Postoperative Delirium in elderly patients following elective Spinal Surgery. Clin. Spine Surg..

[107] Ha A., Krasnow R.E., Mossanen M., Nagle R., Hshieh T.T., Rudolph J.L., Chang S.L. (2018). A contemporary population-based analysis of the incidence, cost, and outcomes of postoperative delirium following major urologic cancer surgeries. Urol. Oncol. Semin. Orig. Investig..

[108] Calderón Rodríguez A., Rodríguez Castaño R., Alonso Marín A. (2018). Delirio En El Paciente Anciano Ingresado En Una Unidad De Cuidados Intensivos: Una Complicación Frecuente.

[109] Steiner L.A. (2011). Postoperative delirium. Part 2: Detection, prevention and treatment. Eur. J. Anesthesiol..

[110] Fukata S., Kawabata Y., Fujishiro K., Kitagawa Y., Kuroiwa K., Akiyama H., Takemura M., Ando M., Hattori H. (2016). Haloperidol prophylaxis for preventing aggravation of postoperative delirium in elderly patients: A randomized, open label prospective trial. Surg. Today.

[111] Rincón Franco I., Cortés Pomar J.F. (2017). Utilidad del Índice Biespectral (BIS) en Delirio Posoperatorio en Pacientes Con Déficit Neurocognositivo de la Fundación Cardioinfantil.

[112] Shankar P., Mueller A., Packiasabapathy S., Gasangwa D., Patxot M., Gara B.O., Shaefi S., Marcantonio E.R., Subramaniam B. (2018). Dexmedetomidine and intravenous acetaminophen for the prevention of postoperative delirium following cardiac surgery (Dexacet trial): Protocol for a prospective randomized controlled trial. Trials.

[113] Koskderelioglu A., Onder O., Gucuyener M., Altay T., Kayali C., Gedizlioglu M. (2017). Screening for postoperative delirium in patients with acute hip fracture: Assessment of predictive factors. Geriatr. Gerontol. Int..

[114] Winter A., Steurer M.P., Dullenkopf A. (2015). Postoperative delirium assessed by post anesthesia care unit staff utilizing the Nursing Delirium Screening Scale: A prospective observational study of 1000 patients in a single Swiss institution. BMC Anesthesiol..

[115] JSmith P., K Attix D., Weldon B.C.G., Monk T. (2016). Depressive Symptoms and Risk of Postoperative Delirium. Am. J. Geriatr. Psychiatry.

[116] Hernandez B.A., Lindroth H., Rowley P., Boncyk C., Raz A., Gaskell A., García P.S., Sleigh J., Sanders R.D. (2012). Post-anaesthesia care unit delirium: Incidence, risk factors and associated adverse outcomes. Br. J. Anaesth..

[117] Järvelä K., Porkkala H., Karlsson S., Martikainen T., Selander T., Bendel S. (2017). Postoperative Delirium in Cardiac Surgery Patients. J. Cardiothorac. Vasc. Anesth..

[118] Vilchis-Rentería J.S., Zaragoza-Lemus G. (2012). Déficit cognitivo en artroplastía de cadera y rodilla. Rev. Mex. Anestesiol..

[119] Stephani Hernández A., Sánchez J. (2014). Factores anestésicos asociados con el deterioro cognitivo postoperatorio en el paciente geriátrico. Rev. Mex. Anestesiol..

[120] Kassie G.M., Nguyen T.A., Kalisch L.M., Nicole E., Roughead E.E. (2018). Do risk prediction Models for Postoperative Delirium consider patients’ preoperative medication use?. Drugs Aging.

[121] Saller T., Hofmann-Kiefer K.F., Saller I., Zwissler B., Von Dossow V. (2021). Implementation of strategies to prevent and treat postoperative delirium in the post-anesthesia caring unit A German survey of current practice. J. Clin. Monit. Comput..

[122] González-Vaquero M., Carriedo-Ule D., Domínguez-Berrot A.M., González-Luengo R., Jiménez-García P. (2015). Complicaciones de la reanimación cardiopulmonar asistida telefónicamente Complications of cardiopulmonary resuscitation telephone assisted. Med. Intensiv..

